# Correction to “Contemporary Perspectives on Chronic Renal Disorders”

**DOI:** 10.1002/cdt3.70049

**Published:** 2026-04-15

1

Ashok D, Manjrekar PA, Shetty BC, et al. Contemporary Perspectives on Chronic Renal Disorders. *Chronic Diseases and Translational Medicine*. 2025;11:89–104, https://doi.org/10.1002/cdt3.70004

In the published article, there were errors in the reference list. References 78 and 79 were duplicated. The correct reference should read:

E. Critselis, A. Vlahou, V. S. Stel, and R. Morton, “Cost‐Effectiveness of Screening Type 2 Diabetes Patients for Chronic Kidney Disease Progression with the CKD273 Urinary Peptide Classifier as Compared to Urinary Albumin Excretion,” *Nephrology Dialysis Transplantation* 33, no. 3 (2018): 441–449, https://doi.org/10.1093/ndt/gfx068

Additionally, references 137 and 138 were duplicated. The correct reference should read:

N. Pinto and M. Eileen Dolan, “Clinically Relevant Genetic Variations in Drug‐Metabolizing Enzymes,” *Current Drug Metabolism* 12, no. 5 (2011): 487–497, https://doi.org/10.2174/138920011795495321

We apologize for this error.

